# Community participation of community dwelling older adults: a cross-sectional study

**DOI:** 10.1186/s12889-021-10592-4

**Published:** 2021-03-29

**Authors:** Claire Gough, Lucy K. Lewis, Christopher Barr, Anthony Maeder, Stacey George

**Affiliations:** 1 College of Nursing and Health Sciences, Flinders University, Sturt Building N219, GPO Box 2100, Adelaide, SA 5001 Australia; 2 Flinders Digital Health Research Centre, Flinders University, Adelaide, Australia; 3 Caring Futures Institute, Flinders University, Adelaide, Australia

**Keywords:** Community participation, Older adults, Global positioning system, Accelerometry

## Abstract

**Background:**

With the advancing age of the population, and increasing demands on healthcare services, community participation has become an important consideration for healthy ageing. Low levels of community participation have been linked to increased mortality and social isolation. The extent to which community participation has been measured objectively in older adults remains scarce. This study aims to describe where and how older adults participate in the community and determine the feasibility of measurement methods for community participation.

**Methods:**

This observational cross-sectional study obtained data from 46 community dwelling older adults. A combination of Global Positioning Systems (GPS), accelerometry, and self-reported diaries were used over a 7-day monitoring period. Feasibility of methods were determined by calculating the loss of GPS data, questionnaires, and comparison of self-reported locations with GPS co-ordinates. Relationships between community participation, physical activity, social interactions, health related quality of life, sleep quality and loneliness were explored.

**Results:**

Older adults took a median (IQR) of 15 (9.25–18.75) trips out of home over the 7-day monitoring period, most frequently visiting commercial and recreational locations. In-home activities were mainly sedentary in nature, with out of home activities dependent on location type. Self-reported and GPS measures of trips out of home and the locations visited were significantly correlated (self-report 15.7 (5.6) GPS 14.4 (5.8) (r = 0.94)). Significant correlations between both the number of trips taken from home, with social interactions (r = 0.62) and the minutes of moderate to vigorous physical activity (MVPA) (r = 0.43) were observed. Daily MVPA was higher in participants who visited local walk/greenspaces (r = 0.48).

**Conclusion:**

Participants performed more activities with social interactions out of home and visited commercial locations most frequently. The combination of GPS, accelerometry and self-report methods provided a detailed picture of community participation for older adults. Further research is required with older adults of varying health status to generalise the relationships between community participation, location and physical activity.

**Trial registration:**

Ethical approval was gained from the Flinders University Social and Behavioural Research Ethics Committee (protocol no. 8176).

**Supplementary Information:**

The online version contains supplementary material available at 10.1186/s12889-021-10592-4.

## Background

With the increasing age of the population and high prevalence of chronic disease, the demands on healthcare systems continue to grow [1]. By 2057, it is estimated that 22% of the Australian population will be aged 65 years and older [2], with costs of ageing expected to rise from $18 billion in 1996, to $24 billion in 2051 [3]. Despite increasing life expectancy, there is no guarantee of ‘healthy’ ageing or quality of life in the latter years [4, 5]. Therefore, the importance of facilitating healthy ageing has increased.

The term ‘community participation’ is defined as ‘*engagement in activities occurring outside the home that are complex in nature, social and nondomestic* [6].’ These activities may include, meeting with family or friends, taking part in recreational activities, volunteering and cultural or social activities [7, 8]. ‘Social participation’ is defined as ‘*a person’s involvement in activities that provide interaction with others in society or the community* [9].’ Older adults who participate in such activities have a lower risk of functional disability, increased health related quality of life (HRQOL) and report lower usage of formal healthcare [10, 11]. Community and social participation are therefore key components of healthy ageing [12] and an important consideration for future healthcare delivery [13].

Participating in the community becomes more difficult with increasing age due to increasing frailty and reduced mobility [6]. In order to remain active out of their homes, older adults need to maintain functional mobility, and overcome personal and environmental barriers [14, 15]. Many older adults become dependent on community resources and planned activities for meaningful engagement and social interactions [16]. Despite the benefits of community participation, the evidence is sparse regarding how and where older people participate in their communities. Understanding these factors mayallow for support and interventions specifically targeting the promotion of participation through the latter stages of life.

Factors that determine community participation are not yet fully understood [17]. However, levels of physical activity (PA) and the preservation of functional mobility is vital for participation in community activities [14]. The World Health Organization defines PA ‘as *any bodily movement produced by skeletal muscles that requires energy expenditure* [18]*.’* Adults aged over 65 years are recommended to engage in at least 30 min of moderate intensity PA, five times per week, or 75 min of vigorous activity per week [19–21]. Despite these guidelines, levels of PA remain particularly low in this age group [22] with reports older people can spend up to 80% of their day sedentary [23]. High levels of sedentary time have been associated with older individuals with chronic conditions and/or walking difficulties [24]. Therefore, increasing or maintaining PA levels allows older adults to participate in and contribute to society, with active community participation in turn leading to increased levels of PA [25].

Furthermore, the social components of activity are important in keeping older adults motivated and engaged, with social isolation and loneliness known to reduce engagement in both the community and PA [26, 27]. Social isolation is defined as an *‘objective lack of relationships and social interaction*’ and loneliness as ‘*a subjective and distressing feeling* [28].’Older adults have an increased risk of social isolation and loneliness stemming from events such as transitioning into retirement [29]. The risks are also linked to poor sleep quality [28], increased blood pressure [30], impaired cognitive function [31] and depression [32]. Social integration of older adults via community participation has been demonstrated to improve quality of life (QoL) [33]. There is a need to explore the factors associated with community participation for older adults, to inform interventions which can maximise QoL and wellbeing.

When developing strategies to increase community participation in older adults, objective measures are required to gain a detailed picture of baseline levels [34]. Despite this, self-reported methods that lack objectivity and often analyse a specific activity rather than daily patterns, continue to be used [34–37]. With advances in health monitoring technology, there is the potential to use objective measures to measure components related to community participation [36]. Previous research has successfully combined Global Positioning Systems (GPS) with accelerometry allowing for the assessment of indoor and outdoor PA, reflecting unstructured activity in a normal day [38]. Previous recommendations have combined objective measures of PA and outdoor time [39]. Accelerometers measure body movement in real-time, specifically the intensity, frequency, duration and total volume of activity [40]. GPS is a satellite based navigation system, which allows receivers to calculate exact locations [41]. It has the potential to be applied to the assessment of community participation in older adults by measuring the number of outings away from home as a representation of community participation [42–44]. The use of GPS combined with accelerometry can determine specific community locations and intensity of activity, despite this, objective reports of community participation for older adults are lacking [45].

Therefore, the primary aim of this study was to describe community participation (specifically by location type and frequency) in community-dwelling older adults and characterise the types of activities in which they engaged. The secondary aim was to investigate the factors associated with community participation, including PA, social interaction, HRQOL, sleep quality and loneliness. The tertiary aim was to determine feasibility of a 7-day monitoring protocol using GPS, accelerometry and self-reported diaries with older adults, and to determine the validity of associated quantitative and qualitative measures.

## Methods

This study used an observational cross-sectional design. Ethical approval was gained from the Flinders University Social and Behavioural Research Ethics Committee (protocol no. 8176). Written informed consent was obtained from all participants. Data were collected from November 2018 to May 2019. Methods combined quantitative and qualitative measures of community participation and physical activity, to provide a data rich picture of participation [46].

### Participants

For inclusion, participants had to live in metropolitan Adelaide, be able to walk independently (+/− walking aids), speak and understand English, have sufficient cognition to understand the study information and be aged 65 years or over. Individuals were excluded if they were living in residential care facilities. Participants were recruited using flyers advertising the study through local Councils, community centres, social media forums and organisations for older adults. Interested individuals were invited to contact the Principal Researcher (CG) who screened potential participants for eligibility over the phone. The Standardised Mini Mental State Examination (SMMSE) was completed with potential participants to determine whether they had sufficient cognitive capacity to participate in the study, with a score above 25 required for participation [47].

### Outcomes

#### Community participation

Community participation was measured using GPS (Qstarz BT1000XT) to calculate the number of trips away from home, type of location visited and the number of in- and out-of-home activities. The Qstarz BT1000XT device is deemed to be accurate to within 10 m for 79% of ≈68,000 GPS points [48] and a popular device with researchers [42]. GPS data provided co-ordinates of the beginning and end locations of identified ‘loops’ for individual trips. The co-ordinates were viewed on the street view of Google maps [49] to identify the location visited. The types of location were then grouped into the following categories: residential, recreational, commercial, health, local walk/ greenspace, central business district (CBD) and place of worship [8] (Table 1). For each type of location visited out of home, activity diaries were cross-referenced to ascertain the purpose of the visit and to identify possible social interactions. For example, in a commercial location, grocery shopping was identified as a domestic task, yet attending a walking group in a shopping centre was deemed an important social component of community participation.

**Table 1:** Community Participation category definitions [8]

Residential	Housing other than own home
Recreational	Sports centre, community hall, swimming pool
Commercial	Shopping centres, local shops
Health	Hospital, GP clinic, physiotherapist, blood clinic
Local walk/Greenspace	Local area, park space (beach), or greenery close to home
Central Business district	Adelaide Central Business District (CBD)
Place of worship	A location designed for congregation of faith

Self-reported participation diaries were completed by participants to provide the context of community participation. Diaries reported the time, activity, duration, location and social interactions out of the home. Participants recorded sleep and device non-wear. An excerpt is provided in Additional file 3. This information was used to cross-check with the objective data, the accuracy of location where GPS data were missing, and report participation in specific activities.

#### Community participation- influencing factors

*Physical activity* was objectively measured with GeneActiv wrist-worn accelerometers, fitted to the non-dominant wrist. GeneActiv accelerometers have been deemed reliable and valid for classifying the intensity of PA in adults [50]. Accelerometer data were used to determine times participants were sedentary, and engaging in light, moderate or vigorous activity. To determine overall daily PA, GeneActiv .bin files were converted to 60-s epoch files and analysed using Cobra software (Francois Frayasse, University of South Australia). Cut points developed by Esliger [50] were used (adjusted for the sampling frequency and epochs) to identify activity intensity (light 283, moderate 605 and vigorous 1697). Sleep was identified using a combination of visual analysis of the activity trace, and self-reported sleep diaries, and subsequently excluded from the analysis.

The *number of social interactions *experienced were self-reported by participants in participation diaries (Additional file 3), with the total number and location of social interactions identified. *HRQOL *was measured using the AQOL-8D questionnaire, deemed valid and reliable with larger samples [40]. AQOL-8D utility algorithm was used to calculate scores [51] which were compared with the general population [52], across the following categories: Independent living (IL), Pain, Senses, Mental health, Happiness, Coping, Self-worth and Relationships, with higher scores indicative of greater quality of life. *Sleep quality* was measured using the Pittsburgh Sleep Quality Index (PSQI), an instrument used to measure the quality and patterns of sleep in older adults [53]. PSQI scores were calculated manually, with scores of 6 and above used to identify poor sleep quality [53]. Levels of *loneliness* were measured using the De Jong Gierveld loneliness scale which has been validated to measure general, emotional and social loneliness [54]. Scores were calculated, with a score of 6 representing ‘most lonely’ (on a scale of 0–6) [54].

#### Feasibility of community participation measurement

Loss of GPS hours were calculated to determine the completeness of data collection, according to the expected number of cells recorded [*n* = 120,960 (5 s epochs)] [44]. The self-reported locations and number of trips out-of-home (total) were manually checked against GPS co-ordinates to determine the accuracy of self-reported location (community participation). Data were linked to Google Maps [49] for graphic representation of where participants were in the community. A maximum time-difference of 10 min was accepted for reported location analysis [55]. On study completion, a 15-item feasibility questionnaire to determine participant experiences of wearing the deviceswas completed [56].

### Procedure

Eligible participants attended a face-to-face meeting with the researcher, either in their own home or at the university. At this meeting, participants completed demographic, AQOL-8D, PSQI and De Jong Gierveld loneliness questionnaires and were measured for height and weight using standardised procedures.

**Fig. 1:**
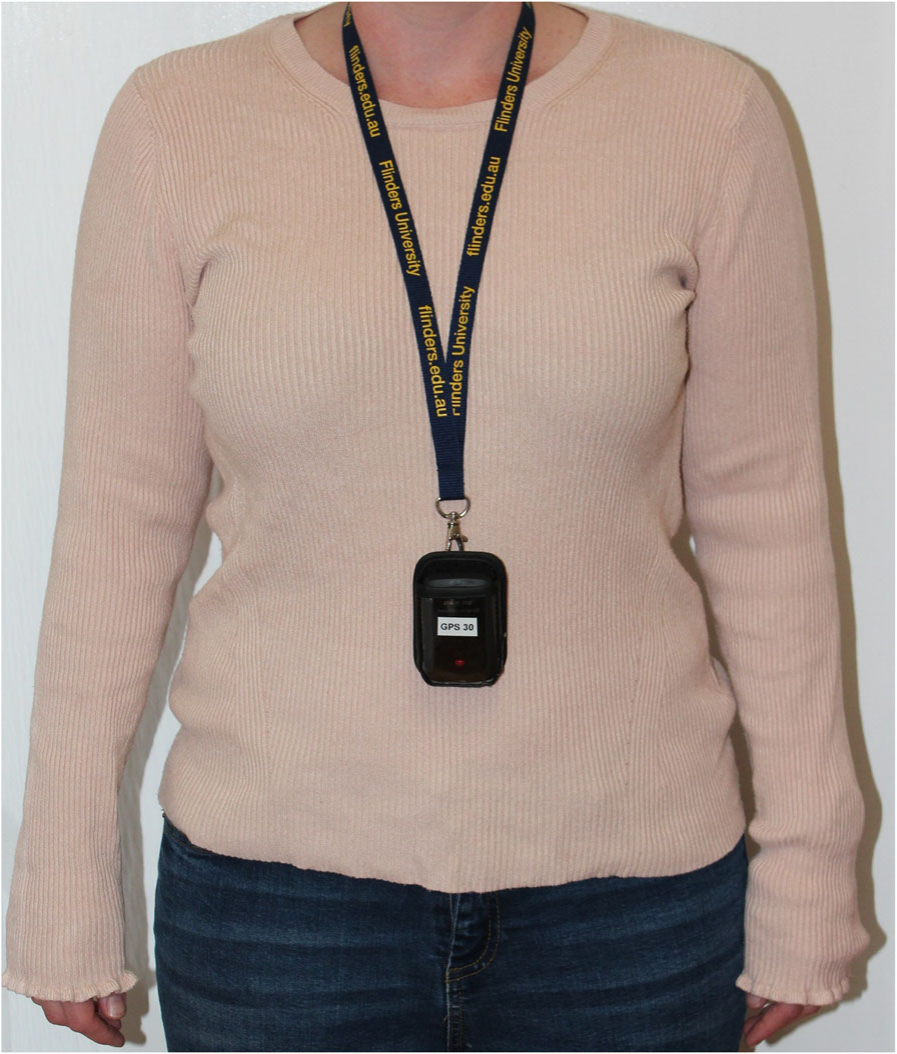
Qstarz BT1000XT GPS device

**Fig. 2:**
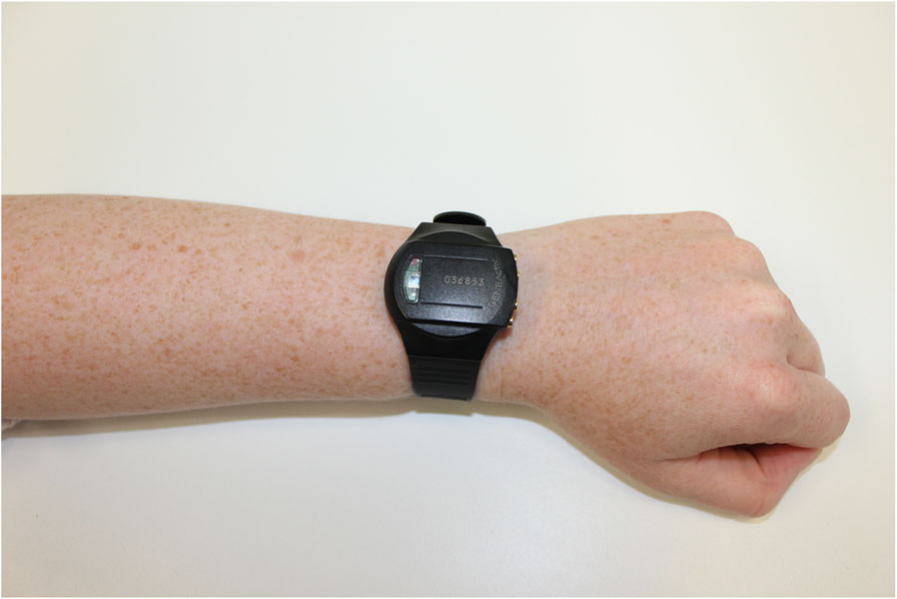
GeneActiv triaxial accelerometer

Participants were fitted with a Qstarz BT1000XT GPS device (Fig. 1) and GeneActiv triaxial accelerometer (Fig. 2) with device instructions. The researcher explained how to use the devices and assisted with setup for each participant. The GPS device was worn on a lanyard around the neck, attached to a belt loop, on a waist belt or in the participant’s pocket (depending on preference and comfort). The device measured 72.2 mm (L) × 46.5 mm (W) × 20.0 mm (H), weighed 8.5 g and had a battery life of 42 h. The GeneActiv accelerometer was fitted comfortably to the participant’s non-dominant wrist. The device measured 43 mm (L) × 40 mm (W) × 13 mm (H) and weighed 16 g.

Participants were asked to wear the GeneActiv device continually for the 7-day monitoring period (inclusive of sleeping, showering and swimming- as the devices were waterproof). GPS devices were to be worn whenever participants left their home and were removed for water-based activities and overnight for charging. Participants were asked to carry out their normal daily routines whilst wearing the devices. Devices were synchronised to begin recording and obtain 7-days of 24-h data, recording at 5-s epochs, with GeneActiv devices recording at a frequency of 75 Hz. Reminder signs were provided to the participants; the first was to be placed near the bed to prompt charging of the GPS device each night, the second was meant to be placed near the exit to the home to prompt participants to take the GPS device with them.

Participants were provided with the option to receive daily reminders to charge the GPS device via text message during the monitoring period. On day three, all participants were contacted via telephone to discuss any issues and to provide a reminder to charge the GPS device. During the monitoring period, participants kept a written diary detailing their activities. On completion of the study, participants attended an exit meeting where the researcher collected the devices and participation diaries. Participants were then invited to complete the feasibility questionnaire.

#### Data processing

Signal loss from GPS devices is a common and well-documented issue [43, 44, 57], therefore quality measures were prospectively determined for the inclusion of data sets in the analysis. To be included, GPS data required a minimum of 8- h (480 min) for each day, complete for five of the seven days monitored [58]. For the accelerometer data, to be included in the analysis, a minimum of four valid days, defined as the recording of at least 8-h of waking time, with at least one weekend day required [59, 60].

For the determination of community participation, GPS data were downloaded as .csv files using QSTARZ DataViewer Version 1.37.000 software [61]. GPS data were cleaned to remove title lines that were recorded when GPS signal had been interrupted. GPS data recorded prior to the start of accelerometry monitoring were also removed. Accelerometry data were downloaded using GeneActiv PC Software version 3.2 as .bin files and converted into 5-s epoch .csv files. GPS and accelerometry files were then combined using time stamps with Python coding software version 2.7.14 [62]. These methods allowed for the detection of when and where participants participated in community activity, following recommendations for proper data handling and maintenance of correct time stamps [63]. Self-reported diary entries were recorded in an Excel spreadsheet, where locations reported were grouped into residential, recreational, commercial, health, local walk/greenspace, CBD and place of worship [44]. Activities such as gardening were noted as in-home activities, and reports of social interaction were identified according to location.

#### Data analysis

Data were entered and analysed using the Statistical Package for Social Sciences (SPSS) [64] with identifying information removed. Questionnaire responses were entered by a researcher and crosschecked by a research assistant. Descriptive analyses were performed for participant demographic data. The normality of data was determined using Z scores [65] with means and standard deviations (SD) reported for normally distributed data, and median and IQR for non-normally distributed data. Spearman correlations were performed to identify the relationships between the number of social interactions, the number of minutes of MVPA, HRQOL, loneliness, and sleep quality scores with the total number of trips away from home and with the number of trips to different locations [66]. Paired t-tests (significance set to alpha of 0.05) were used to determine the accuracy of self-reported location with GPS locations with significance set at < 0.01 for Spearman’s, due to multiple correlations [67, 68].

## Results

### Participant characteristics

*A* total of 46 participants (*n* = 33, 72% female), mean age 74 years (SD 5) participated in the study. The sample demonstrated ‘normal’ cognition [47] with a mean SMMSE score of 29.2 out of 30 (SD 1.3). Thirty-nine percent of participants were married/in de facto relationships and 61 percent were either single, separated or widowed. Participants self-reported an average of two chronic conditions each and all participants reported their general health as good or above (Table 2).

**Table 2:** Participant characteristics

Characteristic	Participants (***n*** = 46)
Gender (M:F) n (%)	13:33 (72:28)
Age mean (SD) years	74 (5)
BMI mean (SD)	28 (5)
Underweight n (%)	0 (0)
Normal n (%)	16 (34)
Overweight n (%)	15 (33)
Obese n (%)	15 (33)
Marital status n (%)	
Single/never married	2 (4)
Separated/divorced	10 (22)
Widowed	16 (35)
Married/defacto	18 (39)
Education level n (%)	
High-school	9 (19)
Post-secondary	16 (35)
Bachelor degree	11 (24)
Post-graduate	10 (22)
Index of Relative socio-economic advantage and disadvantage score (IRSAD) n (%)	
1	4 (9)
2	4 (9)
3	14 (30)
4	15 (33)
5	9 (20)
Employment status n (%)	
Employed	2 (4)
Retired	44 (96)
Volunteer n (%)	28 (61)
No. of volunteer hours per week mean (SD)	4 (7)
Pet owner n (%)	13 (28)
No. of co-morbidities mean (SD)	2 (1)
Self-rated general health n (%)	
Excellent	8 (17)
Very good	25 (54)
Good	13 (28)
Fair	0 (0)
Poor	0 (0)
SMMSE mean (SD)	29 (1)

Standard deviation (SD), Body mass index (BMI), Index of relative socio-economic advantage and disadvantage score (IRSAD) (higher score indicative of lack of disadvantage and greater advantage in general), Standardised Mini Mental State Examination (SMMSE), IRSAD score, low score denotes greater disadvantage and lack of advantage in general

Valid Data sets were obtained for 44 of the 46 recruited participants. Data were excluded from one participant due to equipment malfunction (GeneActiv device). One further data set was excluded as the participation diary was not completed and therefore could not be included in comparisons of self-reported and GPS locations. Following exclusions, of the 7392 h of expected GPS data, 6983 h were recorded. Two participants requested reminders to charge the GPS device and received text messages on days 1, 2, 4, 5 and 6.

### Community participation

**Fig. 3:**
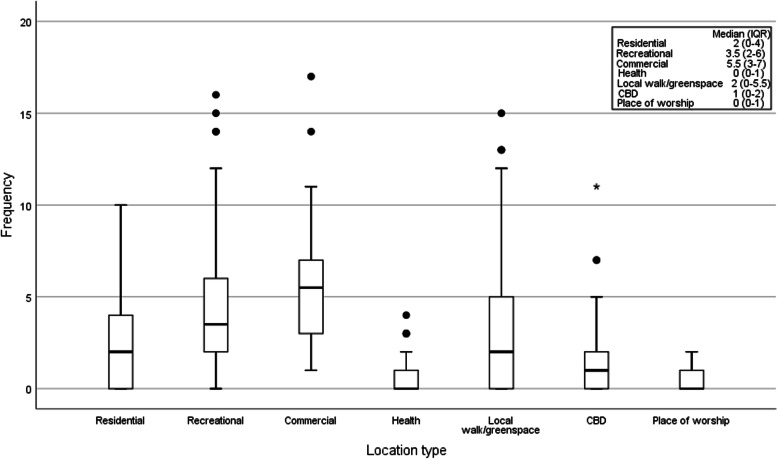
GPS out-of-home activity locations visited over the 7-day monitoring period. (● represents outliers,  represents extreme outlier)

Overall, participants reported a median (IQR) of 15 (8–18) in-home activities and 18 (14–25) out-of-home activities over the 7-day period, with median (IQR) of 15 (9–19) GPS trips out-of-home. Nine participants reported a single day where they did not leave the house. The median (IQR) number of locations visited outside of the home are presented in Fig. 3*,* with commercial locations the most frequently visited location type (median 6, range: 3–7), followed by recreational 4 (2–6), local walk/greenspaces 2 (0–6), residential 2 (0–4), CBD 1 (0–2), health 0 (0–1) and place of worship 0 (0–1).

The type of in-home activities reported are detailed in Additional file 1and were mainly sedentary in nature, including reading the paper, computer work, watching television and listening to the radio. The type of out-of-home activities varied with the location (Additional file 1).

### Community participation- influencing factors

Physical activity varied between participants, with a daily median of 67 min of moderate-vigorous physical activity (MVPA) (IQR 38–89). A daily median (IQR) of 223 (195–294) minutes were spent performing light activity, 65 (36–89) minutes moderate intensity activity and 20 s (0–117) of vigorous activity. Twenty-seven (61%) participants performed no vigorous activity. The mean sleep time was 480 (SD 58) minutes per night andon average, participants spent 659 min (SD 91) per day sedentary. Wear time was examined for each participant by manually reviewing the GeneActiv activity trace for each day of data collection. There was 100% compliance for the 24 h/d, 7d monitoring protocol for the GeneActiv devices (1440 min/d wear time each day for all participants).

**Fig. 4:**
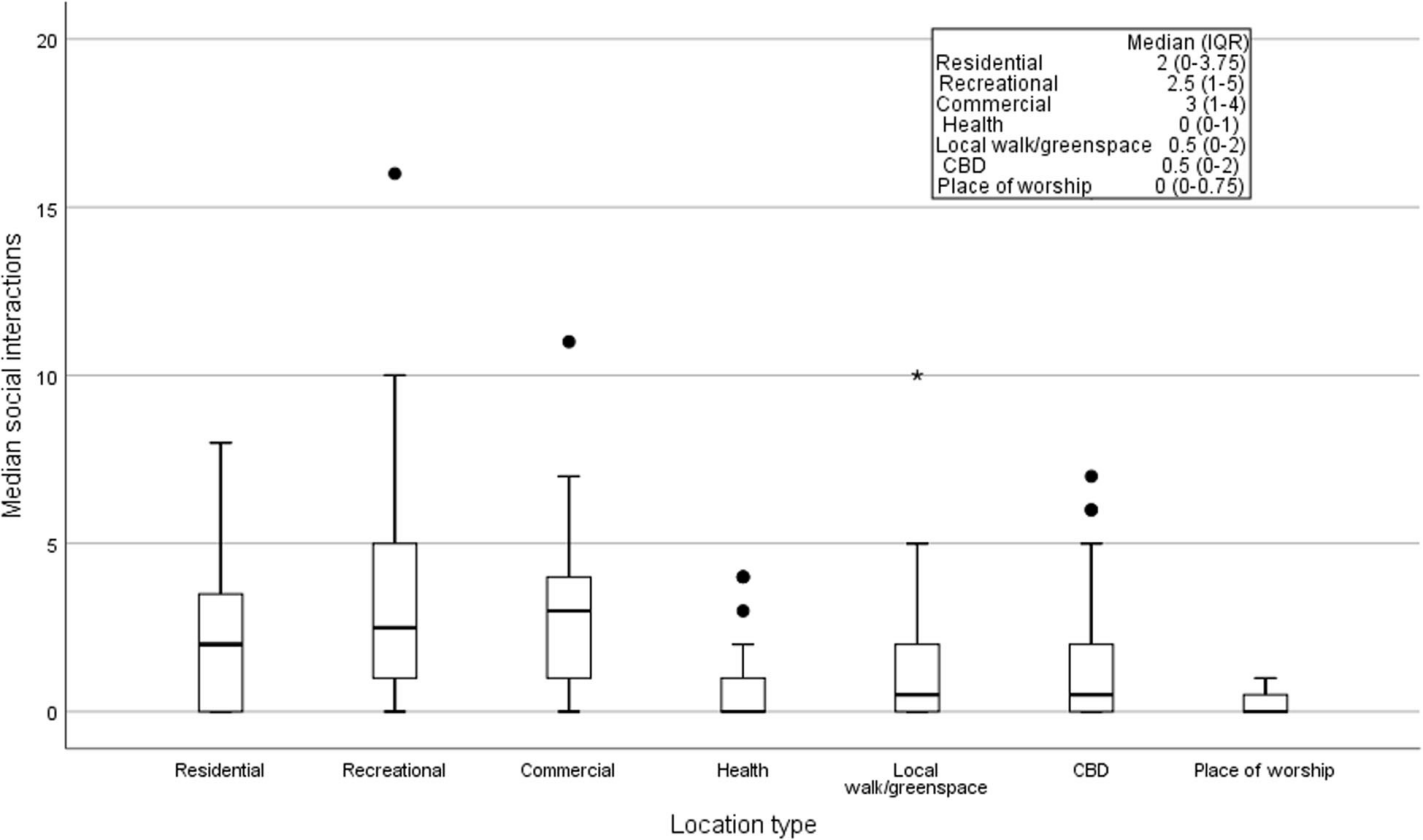
Locations of social interactions over the 7-d monitoring period. (● represents outliers,  represents extreme outlier)

Overall, participants reported a median (IQR) of 2 (0–7) in-home social interactions and 11.5 (8–17) out-of-home social interactions over the 7-days. A median (IQR) of 16.5 (10–21) social interactions were reported over the 7-days (positively skewed 0.24). The median (IQR) number of locations of social interactions over the 7-days is presented in Fig. 4. The most common location type for social interactions was recreational, median (IQR) 3 (1–4) and commercial 3 (1–5) followed by residential 2 (0–4). No social interactions were reported at health, local walk/greenspaces, CBDor places of worship.

The mean (SD) AQOL-8D score for the HRQOL for the general population aged between 65 to 74 years old is 0.83 (0.22) [52], which is matched closely by the participant mean (SD) 0.84 (0.75) in this sample. The study sample reported higher Mental Super Dimension (MSD) scores, mean (SD) 0.84 (0.77), when compared with the general population 0.50 (0.01) which combined mental health, coping, self-worth and relationships [69]. Sleep quality ranged from 1 to 14 on the PSQI with a mean of 5.41 (SD 3) with higher scores indicative of poor sleep quality, 19 (43%) participants scored over 6 [53] indicating poor sleep quality. The study sample detected two participants who scored five out of six on the De Jong Gierveld Loneliness Scale, suggesting feelings of loneliness. The overall mean of the sample was 1.4 (SD 1.4), representing a non-lonely group.

Positive correlations were found between both the trips away from home and social interactions (0.62) and trips away from home and minutes of daily MVPA (0.43) (Table 3). There was a positive correlation between visits to local walk/greenspaces and minutes of daily MVPA (0.48). Increasing age was correlated with reduced minutes of MVPA (0.42). No significant associations were found between trips away from home and HRQOL, loneliness or sleep quality.

**Table 3:** Correlation between the number of social interactions, the number of minutes MVPA, HRQOL, loneliness and sleep quality scores with the total number of trips away from home and with the number of trips to different location types (*n* = 44)

Spearman’s rho	Social interactions	MVPA	HRQOL	Loneliness	Sleep quality
**Trips away from home**	0.615^b^	0.434^b^	0.006	−0.134	− 0.240
**Residential**	0.322^a^	0.133	−0.206	−0.210	0.034
**Recreational**	0.384^a^	0.267	−0.205	0.016	0.114
**Commercial**	0.260	0.118	0.146	−0.144	− 0.272
**Health**	0.142	0.033	−0.133	0.106	0.144
**Local walk/greenspace**	0.196	0.477^b^	−0.076	0.002	−0.204
**CBD**	0.151	0.026	−0.239	0.260	0.095
**Place of worship**	0.144	−0.069	−0.128	− 0.061	0.116

^b^. Correlation is significant at the 0.01 level (2-tailed)

^a^. Correlation is significant at the 0.05 level (2-tailed)

### Feasibility of community participation measurement

Loss of GPS data ranged from 0 to 91 h (0–54%) per participant, after excluding one data set that did not meet the quality standards for analysis, the overall range was 0 to 50 h lost with a mean of 9.3 h (SD 11.8) over the 7-day monitoring period. The responses to the feasibility questionnaire indicated that devices were easy to carry (82%), comfortable to wear (54%), easy to remember to charge (54%) and remember when leaving the house (59%). Participants also reported that the reminder flyers were useful to assist with charging and remembering devices. Participants reported that participating in the study did not impact their normal routine (78%), disrupt sleep (100%) and was not time consuming (89%).

**Fig. 5:**
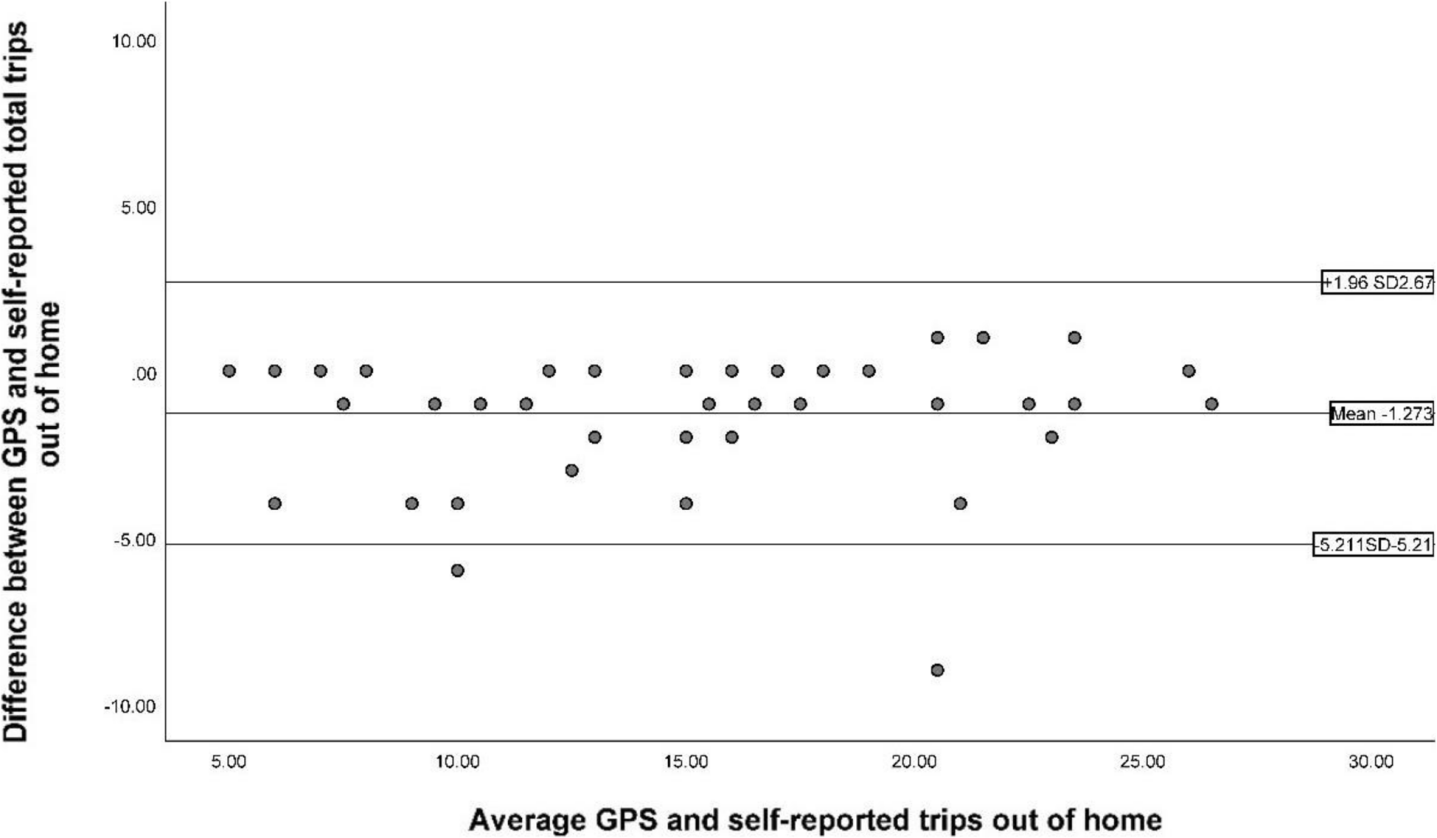
Bland-Altman plot of GPS vs self-reported total trips out of home

To determine whether there were differences between out-of-home self-reported locations and GPS co-ordinates, paired t-tests were performed (Table 4). Participants self-reported a significantly higher number of trips out of the home compared with GPS data (*p* < 0.001). Participant differences between GPS and self-reported trips out of home is provided in Fig. 5. Participants also self-reported a higher number of out-of-home trips to recreational (*p* = 0.005) and commercial (*p* = 0.002) locations than observed in the GPS data (Table 4) (t(43) = 3.284, p = 0.002).

**Table 4:** Difference between self-reported location and GPS location accuracy

Location	Self-report n (mean) (SD)	GPS n (mean) (SD)	Mean difference	T-test	Significance
Total trips out-of-home	15.7 (5.6)	14.4 (5.8)	1.3	4.3	**< 0.001**
Residential	2.5 (2.7)	2.5 (2.6)	0.0	0.0	1.000
Recreational	5.5 (4.2)	4.9 (4.1)	0.6	3.0	**0.005**
Commercial	6.3 (3.4)	5.6 (3.3)	0.7	3.3	**0.002**
Health	0.8 (1.2)	0.7 (1.1)	0.7	1.0	0.323
Local walk/ greenspace	3.8 (4.5)	3.6 (4.4)	0.2	1.8	0.071
CBD	1.7 (2.2)	1.7 (2.3)	0.0	−1.0	0.323
Place of worship	0.2 (.49)	0.3 (.51)	−0.1	− 1.4	0.160

(Bolding denotes significant *p* < 0.05)

## Discussion

This study describes the community participation of older adults living in the community, including the types of activities engaged in, and factors associated with participation. Feasibility of monitoring community participation using both objective (GPS, GeneActiv) and self-reported methods (diary) was also explored. Participants performed more activities with social interactions out of home and visited commercial locations most frequently. Additionally, they were very active in terms of daily MVPA, with visits to local walk/greenspaces positively associated with increased activity. The combination of monitoring methods used in this study was feasible with this group of community dwelling older adults.

The self-reported general health of the older adults in this study was good or better, similar to 70% of older adults in Australia [2]. The participants in this study were active, engaging in over an hour of at least moderate intensity PA per day. Interestingly this is slightly higher than that demonstrated in community dwelling older adults in Germany who recorded 49 (± 39) minutes per day (mean age of 65–89) [70] using triaxial GT3X accelerometers are comparable to the Esliger cut points as per this study analysis [50]. Overall, participants HRQOL scores matched Australian population norms for adults aged 65 and over [3, 52], and demonstrated higher Mental Super Dimension (MSD) scores, which could reflect the health status, independence and social participation demonstrated by the participantsin this study [69].

Participants in our study had a lower sleep quality compared to a Chinese cohort when measured using the PSQI outcome measure [71]. This study sample deemed themselves healthier than the general population and were more active than other samples of older adults, measured with comparable methods [70, 72]. Sample bias could have contributed to the high levels of community participation described in this study. It is more likely that a healthy group would self-nominate for a study measuring community participation and physical activity, than would be experienced by the general population and less healthy groups. Thus, the need for interventions and awareness of the importance of community participation could therefore be more urgent than indicated from this sample.

### Community participation

The majority of reported social interactions experienced out of home, at recreational and commercial locations, may reflect the high numbers of people and interactions required to access services in these locations (i.e. gaining access to leisure facilities through a receptionist). These results suggest older adults participate in more activities and social interactions out of home than in residential settings. Social interaction is important for keeping older adults motivated and engaged [26] and maintaining cognitive function [31]. Services promoting trips to residential and recreational locations could increase social interactions to promote healthy ageing. Interventions providing social interactions for people who are unable to participate in the community are an important consideration to maximise healthy ageing and should also be considered.

Our findings suggest that higher numbers of trips out of home are related to increased MVPA, with visits to local walk/greenspaces inclusive of PA which reflects previous findings [73]. Services promoting visits to local walk/greenspaces could assist with increasing the physical activity levels of older adults. In-home activities were mainly sedentary in nature, with sedentary time high in this active group, accounting for over 10 h per day [74]. These findings agree with previous research where older adults were found to spend 80% (534 min) of their day sitting [56]. Self-reported diaries highlighted activities that participants engaged in between eating an evening meal and going to bed were predominantly sedentary, concurring with previous research that this time of day can be problematic for accruing sedentary time, with television watching commonly occurring during this time of the day [75–77]. Despite participants meeting the MVPA recommendations [19], they spent a lot of time sedentary which suggests there is an opportunity to increase activity and reduce sedentary time even in an active community-dwelling population. Presumably less active older adults with varying levels of health participate in the community far less, which demonstrates the need to increase PA both in and outside of the home.

### Feasibility of community participation measurement

Valid data sets were obtained from all participants except two, with self-reported and GPS locations similar on analysis, providing a detail rich picture of community participation. GPS data loss for this study was approximately 6%, acceptable data loss of 13% has previously been reported with a population of stroke survivors [57]. The compliance with the 24 h, 7d wear protocol with the GeneActiv accelerometer was excellent, with none of the participants removing the device during data collection. The successful retrieval of full data sets could be due to high levels of cognition and motivation to follow the protocol, or the reminders included in the protocol to ensure participant adherence. Participants reported that the flyers were useful as a reminder to charge the GPS device and take the devices when leaving the house. Self-reported diaries provided a backup, to determine location and often provided detailed descriptions of the location and activity performed that could not be interpreted from GPS or accelerometry alone [78].

The number of trips out of home were significantly higher when self-reported than detected by GPS, as were trips to recreational and commercial locations. Differences could be due to short-duration trips taken which were not detected by the GPS device, or signal drop out. Despite being significantly different, the difference in visits to recreational and commercial locations equates to half a trip more over the 7-days, which accounts for the difference in self-reported total visits out of home. Clinically these differences would not be important when considering the planning of interventions. The combination of GPS, accelerometry and self-report was feasible with community dwelling older adults and can provide detailed information.

### Study strengths and limitations

This study has several methodological strengths and limitations. The study used a cross-sectional design so was unable to determine causal relationships of factors contributing to community participation. The study sample was very active and living independently in the community, with all participants capable of walking independently, therefore the results are not generalisable to all older adults. The potential impact of social desirability bias with potential changes in behaviour of participants as they were wearing monitoring devices and knew the aim of the study was to measure community participation, also needs to be considered.

Sleep scores, as well as other variables may have been affected by the extreme temperatures experienced during data collection. Weather conditions have previously been identified as an important consideration when using this methodology [16]. In this study, monitoring occurred over Summer and Autumn in Adelaide, between the 17th of November 2018 and the 10th of May 2019. As a result, 22 participants carried devices on days with maximum temperatures over 35 degrees Celsius. It is possible that the hot weather may have impacted on daily activity, as well as the types and frequency of trips out of the home. Despite strict protocol, we are unable to guarantee that participants carried the GPS devices for the duration of the monitoring period. However, cross checking GPS data against diary entries were performed to reduce this limitation.

Participants self-reported their social interactions which limits the study, as we are unable to be certain that all participants recorded social interaction in the same way. However, as a measure of social interaction, diaries provided details of social experiences and were analysed as best possible. As far as the authors are aware is the first study to combine GPS, accelerometry and self-reported diaries to determine community participation in community-dwelling older adults.

## Conclusion

This study suggests that community dwelling older adults are more socially and physically active out of home. Despite self-reported community participation and GPS locations being similar on analysis, the use of combined methods to provide data rich pictures of community participation is recommended for future studies. This active sample demonstrates the opportunity to increase PA and minimise sedentary behaviour at home, with considerations for both in home activity and community participation required in less active groups to increase PA. Further research is required with other groups of older adults of varying health status (e.g. transitional or residential care) in order to establish possible relationships between community participation, location and PA, in order to design interventions that promote active healthy ageing.

## Supplementary Information

**Additional file 1.** In-home and out of home activities (Sedentary and active).

**Additional file 2.** Process of data analysis.

**Additional file 3.** Participant diary excerpt.

## Data Availability

The datasets analysed during the current study are available from the corresponding author on reasonable request.

## Abbreviations

HRQOL: Health related quality of life
QoL: Quality of life
PA: Physical activity
GPS: Global Positioning Systems
SMMSE: Standardised Mini Mental State Examination
CBD: Central business district
PSQI: Pittsburgh Sleep Quality Index
MVPA: Moderate vigorous physical activity
