# Supplementary material for: Community participation of community dwelling older adults: a cross-sectional study
Source: BMC Public Health. 2021 Mar 29;21:612. doi: 10.1186/s12889-021-10592-4 (PMC8008662; doi:10.1186/s12889-021-10592-4)
Supplement: Supplementary file 1 — Additional file 1. In-home and out of home activities (Sedentary and active). [file 12889_2021_10592_MOESM1_ESM.docx]

**Additional file 1** Participant diary excerpt

**Day 1** (Date- __/__/__ )

| Time | Activity | Duration | Location | Who with? |
| --- | --- | --- | --- | --- |
| 710 | Woke up |  |  |  |
| 1000 | Ballroom dancing | .2.5 hour | (Name redacted) community centre | Friends |
| 12noon | Lunch |  |  |  |
| 1300 | Sewing | 2hours | Home | Alone |
| 1630 | Dinner and TV watching |  | Home | Alone |
| 1900 | Ballroom dancing | 3 hours | (Name redacted) community centre | Friends |
| 2320 | Bed |  | Home | Alone |

**Sleep** Time woke up from sleep (in the morning) 710 .

Times of sleep during the day: Time N/A Duration N/A .

Time N/A Duration N?A .

Time went to sleep (at night) Time 2320 .

**Times of non- wear** (device was taken off): Time N/A . Duration N/A
