# Supplementary figures and images for: Community participation of community dwelling older adults: a cross-sectional study
Source: BMC Public Health. 2021 Mar 29;21:612. doi: 10.1186/s12889-021-10592-4 (PMC8008662; doi:10.1186/s12889-021-10592-4)

**Additional File 2**-

**
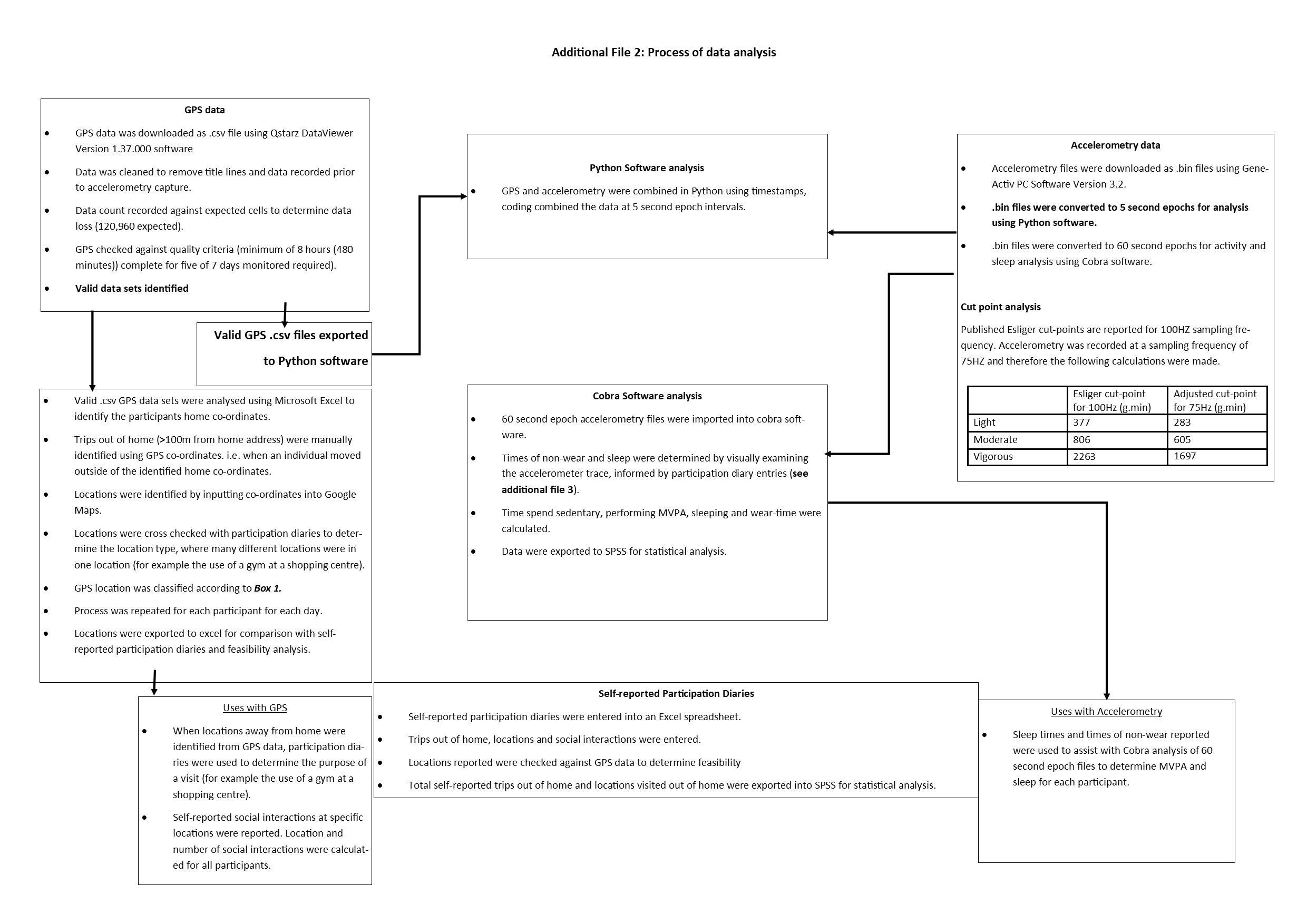
**

Supplement: Supplementary file 2 — Additional file 2. Process of data analysis. [file 12889_2021_10592_MOESM2_ESM.docx]
