# Supplementary material for: Community participation of community dwelling older adults: a cross-sectional study
Source: BMC Public Health. 2021 Mar 29;21:612. doi: 10.1186/s12889-021-10592-4 (PMC8008662; doi:10.1186/s12889-021-10592-4)
Supplement: Supplementary file 3 — Additional file 3. Participant diary excerpt. [file 12889_2021_10592_MOESM3_ESM.docx]

**Additional file 3** In-home and out of home activities (sedentary and active)

| ***In-home activities*** | |
| --- | --- |
| **Sedentary activities reported** | **Active activities reported** |
| Read the paper, computer work, watching television, listening to radio, writing letters, relaxing, napping, phone calls, painting, socialising, friends and/or family visiting, sewing, crosswords, cooking, admin, taking medications, playing on iPhone/iPad, having a haircut, making jewellery, puzzles, volunteer work (admin), knitting, playing the piano and meditating. | Gardening, domestic chores, feeding pets, tidying, watering plants, bowling practice, exercises (following a DVD/You Tube), playing with grandchild, playing with kitten, house maintenance, renovations and preparing for dinner party. |
| ***Out of home activities*** | |
| **Residential activities** | |
| **Sedentary activities reported** | **Active activities reported** |
| Visiting family, visiting friends/for coffee, dinner with friends, attending retirees club meeting, waiting for building inspector, afternoon tea with neighbour, pick up granddaughter, delivering things for neighbourhood watch, meal with family, lunch with friends, having a haircut, acting as Justice of the Peace, tutoring, jewellery making, watching phone technician, meeting with friend, visiting friend in care home. | Assisting client (volunteer work), looking after grandchildren, maintenance of rental property, housework and domestics for son, laundry at friend’s house, feed daughters’ cat and visit daughter to help with housework. |
| **Recreational activities** | |
| **Sedentary activities reported** | **Active activities reported** |
| Visited the library, art gallery visit, had lunch at the golf club, attended a meeting at the golf club, took grandson to basketball, returned library books, had a music lesson, attended a seniors meeting, neighbourhood watch dinner, scrabble group, book club, festival theatre show, volunteer at the theatre, U3A group (University of the third age), Fringe volunteer induction, quiz night, went to the movies, watching tennis, volunteered at the art gallery, played the pokies, Majhong (board game), computer education session, attended COTA group (Older Australians group), attended writers week, watched the ballet, floral art session, attended a wedding, choir rehearsal, art group, computer work at the library, retirement club, attended seniors on screen, training for St Johns Ambulance and attended science group | Walked to library, delivered butterflies to wildlife park, golf, picked up grandchildren from school (walk), gym, ballroom dancing, line dancing, aqua, cricket, tennis, walked around museum, belly dancing class, body balance, strength for life, Pryme movers group, yoga, live music dance, volunteer at Animal Welfare, swim, play lawn bowls, exercise class, table tennis, walked around the zoo, volunteered at the zoo (walking), Pilates, spinners group, Zumba, scouts group, circle dancing, active ageing program. |
| **Commercial activities** | |
| **Sedentary activities reported** | **Active activities reported** |
| Appointment at the bank, pay accounts, went through car wash, eating out, have coffee, eat at McDonalds and read the paper, visit recycling centre, lunch with friends, dinner out, took car for service, visit hairdresser, pick up wife from airport, and charity lunch. | Shopping, fill up with petrol, supermarket shopping, laundromat, garden shop, took client shopping (volunteering), visited post office, visit chemist, walk to shops, walk around DIY shop, ‘op’ shopping, working in studio, window shopping (walking), picked up fish and chips, walked around flower market and plasma donation. |
| **Health activities** | |
| **Sedentary activities reported** | **Active activities reported** |
| Physiotherapy appointment, podiatrist appointment, chemist appointment, doctor’s appointment, XRAY at hospital, blood tests, hospital appointment, dentist, eye test, massage, chiropractor, flu shot and participated in clinical trial at the Hospital. | Hydrotherapy rehab, gym strengthening (rehab) session and St John’s first aid training |
| **Local walk/greenspace activities** | |
| **Sedentary activities reported** | **Active activities reported** |
| None reported | Walked to library, shop, in park, walking, walked dog, walk along beach, walked to beach and back, walked to visit friend, walking group, Park run 5k walk, walk to bus stop, walk to tram, walk to get coffee, morning, afternoon and evening walks, bike ride, walked to gym and walked home |
| **CBD activities** | |
| **Sedentary activities reported** | **Active activities reported** |
| Piano recital, watched cricket, city meeting, dinner, Proms concert, Festival Theatre show, Fringe show, attend writer’s week event, massage, watched ballet and attended Seniors group | Took grandchild to school, attended the Pageant, trip to city, Central markets visit, visited art gallery, voluntary work, Seniors group activity, shopping and walked around the zoo |
| **Place of worship activities** | |
| **Sedentary activities reported** | **Active activities reported** |
| Watched choir concert, dinner, funeral, lunch and attended service. | Active ageing program |
